# Experiences who are d/Deaf and queer individuals: a systematic review and meta-synthesis

**DOI:** 10.1093/jdsade/enaf067

**Published:** 2025-11-19

**Authors:** Kiara Murphy, Sophie C Dahlenburg

**Affiliations:** The University of Adelaide, School of Psychology, North Terrace campus, Adelaide, SA 5000, Australia; The University of Adelaide, School of Psychology, North Terrace campus, Adelaide, SA 5000, Australia

## Abstract

The current systematic review and meta-synthesis investigated the experiences of Deaf queer people, specifically, how identities in this population shift dynamically in response to their dual marginalization. Data were extracted from 27 qualitative studies, with a sample total of 176 participants. Synthesis was influenced by the Model of Multidimensional Identities to frame resulting categories and themes. Four categories were developed from the data: (1) institutional contributors to identity salience, (2) cultural moderators of institutional neglect, (3) resistance to double exclusion through community, and (4) personal navigation of a contested body. The findings capture a picture of complex discrimination, where identities are largely shaped by society’s preconceived prejudices, community, and language access.

## Deaf identity

Identity can be constructed and operationalized as a collective culture or movement, behaviors, attributes, or a quest for belonging within a community (McIlroy & Storbeck, 2011). Identity is multifaceted, as individuals make sense of themselves throughout, and despite, multiple systems of oppression or privilege. Discourse surrounding Deaf identity is long standing within the Deaf Studies discipline and has been adopted within social and cultural psychology. For this study, it is important to make a distinction between Deafness and deafness. The capital D has been an indicator of cultural identification and a rejection of “impairment”, as opposed to a medically affiliated lowercase d deafness (Chin et al., 2013). The capital D Deaf person embraces Deaf Gain (the antithesis of hearing loss) and Deafhood as a source of pride, authenticity, agency, political power, and recognition (Bauman & Murray, 2014; Fernandes & Myers, 2010; Ladd, 2003; Moroe, 2019). Deafness is a cultural anchor that has become a political priority for Deaf activism, representing engagement with community, language, and an acceptance of Deafness as a cultural identity as well as a disability (Beckner & Helme, 2018; Ohna, 2004).

The current second-wave of deaf identity politics, however, acknowledges that rather than a yes/no, binary, d/Deaf people assert themselves on a spectrum, focusing on DEAF-SAME (Kusters et al., 2017; McIlroy & Storbeck, 2011). In response to this second wave, McIlroy and Storbeck (2011) posit a DeaF identity, which answers the call for an acknowledgement of bilingual and bicultural d/Deaf people (i.e., the capital F represents fluidity). DeaFness goes beyond essentialist binaries to acknowledge a postmodern construction of identity, where change and evolution over time is welcomed and intersects with other facets of identity (McIlroy & Storbeck, 2011).

### Multiple identities

While acknowledging the community power of Deafness, prioritizing Deafness as a singular identity risks ignores additional power imbalances (e.g., matrices of oppression) which can enforce “endogenous colonialism” (Wrigley, 1996, pg. 7). When endogenous colonialism occurs, the political Deaf performance assumes to speak on behalf of universal d/Deaf experiences (Smiler & McKee, 2007). For example, research investigating the intersection of Deaf and Māori culture reported that participants’ identity categories felt different, but inseparable (Smiler & McKee, 2007). However, there has been a documented dual exclusion, where Deaf communities do not accommodate for religious or cultural expression, and similarly, religious or cultural communities are not accessible for their d/Deaf patrons, excluding d/Deaf minorities from cultural traditions and knowledge (Dively, 2001; Skelton & Valentine, 2003). This double bind can also lead to discrimination from d/Deaf majority communities, and hearing minority communities (Cohen et al., 1990; James, 2000). Research on racial minorities also highlight the complexity of identity; Foster & Kinuthia’s (2003) study interviewed Asian, Hispanic, and African American d/Deaf students, finding that multiple identities were often constellations, changing over time and setting, meaning different things for each participant.

### Queer identities

Referring to people who are not straight or cisgender within academic research has evolved as the discipline has grown, and discourse surrounding best practice continues (Glazier, 2014). Various acronyms have been operationalized, attempting to clearly define who is included (and not included) in each description (LGBTQIA+, MSM, GSD^1^^;^  Glazier, 2014). For the purposes of this paper the term queer refers to participants who are gender and sexually diverse, acting as a label that encompasses the “historically-contingent and socially-constructed” nature of bodily-desire and experience, rejecting discrete categories (Giffney, 2016, pg. 2). According to Giffney, academic use of the word queer finds strength in its opacity, where readers are invited to question the hazy boundaries of identity categories, demanding self-reflexivity.

### Deaf queer identities

The experiences of d/Deaf queer people have been documented steadily, increasing in quantity since the 1970s, and have proliferated scholarly Deaf Studies. These two identities have parallel histories, where the nineteenth-century drive to categorize and exclude, ratifying normalcy, anticipated the creation of the homosexual, and the deaf mute (Cleall, 2015; Foucault, 1978). Prior to this, sexual acts were considered as behavior rather than markers of identity, and hearing ability was not considered a social classification (Cleall, 2015; Moores, 2010). Following this categorization, scientific scrutiny and medicalization was inflicted on both groups: imparting crude cures for both (Cleall, 2015; Drescher, 2010; Gallaudet, 1881). These constructed identities still exist; though they are now operationalized as a source of pride and inherent to one’s position as a social subject.

Today, d/Deaf queer people navigate a world of compulsory heterosexuality and audism (Miller & Clarke, 2020). d/Deaf queer people report higher depressive symptoms, anxiety, poorer quality of life, higher likelihood of cancer diagnosis and chronic lung disease compared to their hearing, cisgender, and heterosexual counterparts (Miller et al., 2019; Mousley & Chaudoir, 2018). These outcomes reflect a severe lack of quality and accessible health education, likely impacted by generational trauma and discrimination (Miller & Clarke, 2020). Somewhat contradictorily, research has shown higher positive self-image and parallel life satisfaction in the d/Deaf gay participants, compared to hearing gay counterparts (Caricato et al., 2020; Swartz, 1995). The d/Deaf community can be a place of love and acceptance. Its insular nature can provide cushioning against isolation, while strong bonds can decrease likelihood of disownership of queer people once they come out (Langholtz & Rendon, 2019).

Deaf people with Deaf parents have been found to have the most accepting mothers upon coming out, compared to hearing and hard-of-hearing participants with hearing parents (Swartz, 1995). Zangas (2005) reported that heterosexual d/Deaf people had overall positive attitudes toward gay and lesbian d/Deaf people and there was a modest correlation between minority race and homophobia, where non-white participants were less homophobic. This finding supports the concept of mutual oppression fostering acceptance (Zangas, 2005). However, transparency and information sharing are culturally valued within the Deaf community, which, coupled with homophobic stigma, may influence experiences of exclusion within the community (Gutman & Zangas, 2010; Kane, 1994).

### The model of multiple dimensions of identity

The model of multiple dimensions of identity (MMDI) is an apt framework to explore the intersecting identities of Deafness and queerness, responding to previous models that consider only unidimensional aspects of identity by proposing a theory of socially constructed identities that are influenced by changing contexts (Jones & McEwen, 2000). The MMDI describes a core self, not associated with identity-facets. An identity’s closeness with the core, however, represents familiarity and comfort. This is juxtaposed with an outside self, which comprises labels and characteristics that are attributable by strangers (Jones & McEwen, 2000). It is also specific regarding the relationship of identities with each other, and how the proximity of identities to the core changes fluidly throughout the lives of each individual (Jones & McEwen, 2000). The three stages of development are Formulaic (no self-perceived connection between multiple identities), Transitional (integration of multiple identities, but frustration with labels and limitations of categories), and Foundational (consistent identity enactment and resistance of stereotypes; Abes et al., 2007). The MMDI’s ability to account for identities shifting and changing over time is integral, given that both queer and d/Deaf identities are subject to personal introspection and social influence. d/Deafness can be genetic, or acquired, and one’s identification with that label can be a lifelong journey (Bat-Chava, 2000). Both are often influenced by integration with their respective communities. The experiences of people who identify with both d/Deafness and queerness can be structured using the MMDI, weaving together stories of proximity to one’s core.

Much of the published literature regarding d/Deaf queer populations have stated the paucity of scholarship on this intersection (Moreman & Briones, 2018). This study aims to translate, synthesize, and interpret current research literature about the experiences of d/Deaf queer people through systematic review, using the MMDI as an analytical framework to interpret emerging categories and themes. It is expected that due to the co-occurrence of multiple matrices of oppression, audism, and homophobia will be intertwined as well as discussed separately as discriminatory experiences. This will be the first systematic review on the topic, producing a novel insight and furthering the call for d/Deaf Studies to be more inclusive, shifting away from a d/Deaf majority perspective that ignores intersecting identities (Fernandes & Myers, 2010).

## Method

The current utilized both a systematic review and qualitative meta-synthesis methodologies to examine the experiences of d/Deaf queer individuals. Qualitative content was gathered from four databases following the Preferred reporting items for systematic reviews and meta-analyses (PRISMA) guidelines, providing a framework to identify relevant studies (Liberati et al., 2009).

### Search strategy

PsycINFO, Embase, Scopus, and Web of Science databases were searched to extract articles for the meta-synthesis. The following Boolean search string was used for keyword searches: “‘Deaf’ OR ‘Sign language’ OR ‘Hearing loss’ OR ‘Hearing disorder’ OR ‘Cochlear implant’ OR ‘Deaf gain’ OR ‘Deaf Identity’) AND (Queer^*^ OR ‘LGBT^*^.exp’ OR ‘Bi?sexual^*^’ OR Lesbian^*^ OR Gay^*^ OR Homosexual^*^ OR Trans?gender^*^ OR Trans?sexual^*^ OR ‘Gender identity’ OR ‘Sexual orient^*^’”. Grey literature was also searched using Google Scholar and included studies’ reference lists (Siddaway et al., 2019).

### Selection criteria

Studies were included in the systematic review if they (1) related to the d/Deaf queer experience; (2) were qualitative in nature (i.e., data from the studies were words and not numbers such as interviews written responses etc.); and (3) comprised participants that were d/Deaf and queer. Studies that discussed intersectionality in a broader sense (e.g., race, class, religion) were included, but only where discussion of the relationship between d/Deafness and queerness was clearly identifiable. Mixed methodology research was also included; however, only qualitative data were analysed.

Inclusion was decided on a case-by-case basis for other forms of literature such as theses and book chapters, using the same Boolean search string on Google Scholar. This subject is under researched, therefore Grey or unpublished literature in the form of dissertations and book chapters are a source of relevant information to provide as much of a holistic analysis as possible (Paez, 2017). Relevant postgraduate dissertations were included due to their relatively rigorous review process. Book chapters were only included where they met quality standards and were peer-reviewed. Studies that (1) did not investigate the experiences of d/Deaf queer people, (2) were solely quantitative, (3) and did not have d/Deaf queer participants were excluded. Two included studies were originally written in Spanish and Italian, and translated by Google Translate, a decision made in alignment with the benefits (accessibility and translational improvement) outlined by Groves and Mundt (2015), and to increase variation in geographical location of included papers.

### Screening process

**Figure 1:**
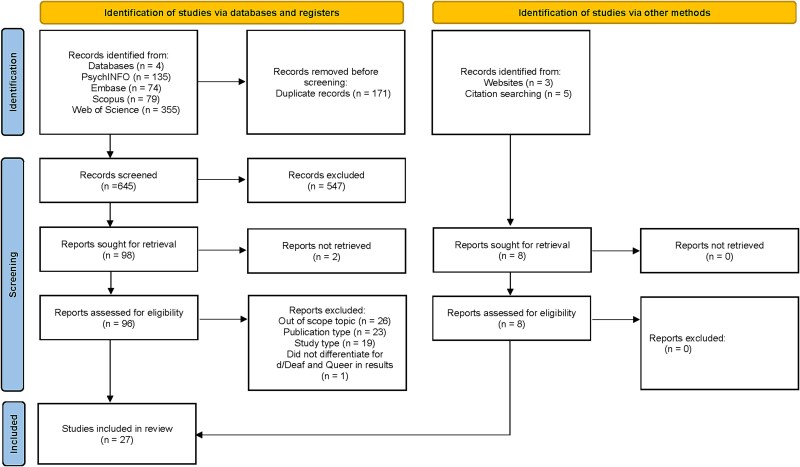
PRISMA flow diagram of study selection.

In total, 816 studies were imported for screening into Covidence, where 171 duplicates were excluded. Six hundred forty-five title and abstracts were then screened, and 98 remained as a result. These studies were screened in full, and 27 were included for data extraction and analysis. Of the 27 included studies, there were ten dissertations, one book chapter, and 13 journal articles. Figure 1 details the screening process.

### Quality assessment

The Joanna Briggs Institute (JBI)’s Instrument of Critical Appraisal Checklist for Qualitative Research was used to assess the quality of the included items. Table 1 details the answers for each included study. For items six and seven, the lack of reflexivity statements had a negative effect on the overall appraisal of the studies. The included dissertations were more likely to have a clear reflexivity statement than the journal articles. The dissertations were less likely to have information about stated ethical approval. Three studies with the highest amount of “No” and “Unsure” answers were due to their primary focus on policy and date of publication. The study by Gomez and Geneta (2021) was still included because of its Asian perspective and focus on young adults. The studies by Phaneuf (1987) and Zakarewsky (1979), while less transparent about methods, are important as forebears of information, were cited by most included articles, and as such are considered pioneering texts.

**Table 1:** Quality assessment of included studies.

	Item 1	Item 2	Item 3	Item 4	Item 5	Item 6	Item 7	Item 8	Item 9	Item 10
	Is there congruity between the stated philosophical perspective and the research methodology?	Is there congruity between the research methodology and the research question or objectives?	Is there congruity between the research methodology and the methods used to collect data?	Is there congruity between the research methodology and the representation and analysis of data?	Is there congruity between the research methodology and the interpretation of results?	Is there a statement locating the researcher culturally or theoretically?	Is the influence of the researcher on the research, and vice- versa, addressed?	Are participants, and their voices, adequately represented?	Is the research ethical according to current criteria or, for recent studies, and is there evidence of ethical approval by an appropriate body?	Do the conclusions drawn in the research report flow from the analysis, or interpretation, of the data?
Beese & Tasker, 2022	Yes	Yes	Yes	Yes	Yes	Yes	Yes	Yes	Yes	Yes
Cappotto & Rinaldi, 2016	Yes	Yes	Yes	No	No	No	No	Yes	Unclear	Yes
Celebrado, 2022	Yes	Yes	Yes	Yes	Yes	Yes	Yes	Yes	Unclear	Yes
Cherasaro, 2018	Yes	Yes	Yes	Yes	Yes	Yes	Yes	Yes	Yes	Yes
Cheslik & Wright, 2021	Yes	Yes	Yes	Yes	Yes	No	No	Yes	Yes	Yes
Conyers, 2008	Yes	Yes	Yes	Yes	Yes	Yes	Yes	Yes	Yes	Yes
De et al., 2015	Yes	Yes	Yes	Yes	Yes	No	No	Unclear	Yes	Yes
Dunne, 2014	Yes	Yes	Yes	Yes	Yes	Yes	Yes	Yes	Yes	Yes
Eckhardt, 2006	Yes	Yes	Yes	Yes	Yes	No	No	Yes	Yes	Yes
Gomez & Geneta, 2021	Unclear	Yes	Yes	Unclear	Unclear	No	No	Unclear	Yes	Unclear
Grant, 2008	Yes	Yes	Yes	Yes	Yes	Yes	Yes	Yes	Yes	Yes
Kelly & Atcherson, 2011	Unclear	Yes	Yes	Yes	Yes	Yes	No	Unclear	Unclear	Yes
Klein, 2021	Yes	Yes	Yes	Yes	Yes	Yes	Yes	Yes	Yes	Yes
Mallinson, 2004	Yes	Yes	Yes	Yes	Yes	No	No	Yes	Yes	Yes
Mauldin & Fannon, 2021	Yes	Yes	Yes	Yes	Yes	Yes	Yes	Yes	Yes	Yes
Mauldin, 2018	Yes	Yes	Yes	Yes	Yes	Yes	Unclear	Yes	Unclear	Yes
Michaels, 2015	Yes	Yes	Yes	Unclear	Yes	Yes	Yes	Yes	Yes	Yes
Miller, Biskupiak, & Kushalnagar, 2019	Yes	Yes	Yes	Yes	Yes	Yes	Yes	Yes	Unclear	Yes
Pfau et al., 2021	Yes	Yes	Unclear	Unclear	Yes	Yes	No	Yes	Yes	Yes
Phaneuf, 1987	Unclear	Unclear	Unclear	Unclear	Unclear	No	No	Unclear	Unclear	Yes
Schaad, 2015	Yes	Yes	Yes	Yes	Yes	Yes	No	Yes	Yes	Yes
Schmitz, 2021	Yes	Yes	Yes	Yes	Yes	Yes	Yes	Yes	Yes	Yes
Sinecka, 2008	Unclear	Yes	Unclear	Yes	Yes	Yes	No	Yes	Unclear	Yes
Ward, 2016	Yes	Yes	Yes	Yes	Yes	Yes	Yes	Yes	Yes	Yes
Whalen, 2014	Yes	Yes	Yes	Yes	Yes	Yes	Yes	Yes	Yes	Yes
Willemse et al., 2009	Yes	Yes	Yes	Yes	Yes	No	No	Unclear	Unclear	Yes
Zakarewsky, 1979	Unclear	Unclear	Unclear	Unclear	Yes	Yes	No	Unclear	No	Yes

### Data analysis

This study adhered to interpretative methods and took a meta-ethnographic approach, where aggregation was not the goal, but translation and the making of new insights and/or conclusions about the data (Noblit & Hare, 1999). Data analysis began with paper collection during the full-text screening phase. Constant comparison can be likened to Noblit and Hare’s (1999) idea of translation into one another, that is, treating each account as an analogy: “One example is like another, except …” (pg. 111). This method documented key ideas, metaphors, phrases, ideas, and concepts to inform eventual themes and categories, maintaining the central ideas of each paper and compared those with the other key concepts found elsewhere, using this constant comparison to create a whole which is more than the sum of its parts (Noblit & Hare, 1999). Re-reading included studies using NVivo 12 facilitated inductive code generation, where focused codes were distilled into themes, and categories. Finally, the MMDI framed the eventual metaphors and themes, giving structure and an analysis framework to the inductive process of code, theme, and category creation.

### Positionality statement

Given the profound effect on writing, it is imperative to state the positionality of both authors, in alignment with Graham and Horejes’ (2017) work on positionality in d/Deaf studies. The first author is a hearing grandchild of Deaf adults, and a lesbian woman. The first author’s experiences as a queer person in the d/Deaf community have motivated her to research this intersection of identities. The first author is a qualified Auslan interpreter and works in d/Deaf education and community events. The first author’s life experiences have been shaped by her Deaf family and their community, allowing her insight and care for the topic, however, her hearing status will forever moderate the depth of analysis able to be achieved by an honorary, that is, not-deaf, community member. The first author carefully self-reflected and collaborated with the second author, considering her experiences and unpacking their meaning, combining both insider and outsider contributions (Graham & Horejes, 2017). The second author has limited exposure to the Deaf community, but conducts research within the queer space, and identifies as a hearing, pansexual, cisgender woman. The analysis and interpretative nuance are inevitably impacted by the absence of lived experience for both hearing authors. Every effort was taken not to influence the resulting findings, represented by the embedding of critical discussion into the research process and centring Noblit and Hare’s reflexive methodological approach to thematic analysis (1999). However, the possibility that the authors’ beliefs and biases, specifically the intention to contribute to the literature in a way that does not perpetuate a deficit-model view of the d/Deaf community, may have influenced the writing of this study.

## Results

### Study characteristics

Twenty-seven studies were included with nine focusing on the experiences of d/Deaf gay men, five focusing on lesbians, one focusing on transgender men, and 12 broadly exploring queer identities. Most studies focused on Deaf identities, however, four studies included Hard of Hearing or bi-lingual perspectives. From the 27 studies there were 176 participants with ages ranging between 18 to 91; however, some studies did not specify ages or only provided decade of birth. The studies comprised participants mostly from the United States (16), but also the United Kingdom (2), Italy (1), the Philippines (2), Brazil (1), the Netherlands (1), Canada (1), Germany (1), Czech Republic (1), and South Africa (1). Studies were published between 1979 and 2022. A full description of study characteristics can be found in Table 2.

**Table 2:** Characteristics of the studies included in the meta-synthesis.

Study information			Participant information						Methodological approach			
Authors, year of publication	Country	Aim of study	Number of participants	Age	d/Deaf identity	Queer identity	Sign language use	Participant’s family composition	Theoretical approach	Sampling	Data collection	Data analysis
Beese & Tasker, 2022	United Kingdom	To widen the consideration of intersecting queer and deaf identities.	8	28–41	Deaf	Gay	4/8 Proficient sign language users	3/8 Deaf family, 5/8 hearing family	Intersectionality theory	Snowball	Interviews	Interpretative Phenomenological Analysis (IPA)
Cappotto & Rinaldi, 2016	Italy	Identifying key arguments which could form the basis for possible future strategic programs.	15	Average age 24.5	Deaf	6 Lesbian 6 Gay 3 Bi-sexual	Information not available	Information not available	Information not available	Snowball	Questionnaire, interviews, online chat	Information not available
Celebrado, 2022	Philippines	Document and examine the Deaf gay men workers’ experiences of discrimination in the private sector.	7	Information not available	Deaf	Gay	Information not available	Information not available	Gender and development theory (GAD), intersectionality theory	Purposeful	Written and face-to-face interviews	IPA
Cherasaro, 2018	United States	Expand the literature base through the use of narrative inquiry conducted with an individual who self identifies as a member of the Deaf lesbian culture.	1	Information not available	Deaf	Lesbian	Proficient sign language user	Hearing family	Crip theory, feminist theory	Purposeful	Interviews	Auto-ethnography
Cheslik & Wright, 2021	United States	Uncover how Gay Social Networking apps are used among the Deaf gay community.	7	24–65	Deaf Hard of Hearing	Gay Queer Pansexual	7/7 Proficient sign language users	Information not available	NA (grounded theory)	Snowball	Interviews	Grounded theory
Conyers, 2008	United States	To investigate the consequences of labeling and management techniques of individuals who possess multiple spoiled identities.	10	18–42	Deaf	6 Gay 3 Lesbian 1 Bi-sexual	Information not available	Information not available	Spoiled identities	Snowball	Online chat interviews	Grounded theory
De et al., 2015	Brazil	To investigate the narratives of deaf people in their first homosexual experiences, emphasizing the double prejudice; being deaf and gay.	3	32–38	Deaf	Gay	3/3 Proficient sign language users	Information not available	Information not available	Convenience	Interviews	Information not available
Dunne, 2014	United States	To explore the lived experiences of three Deaf/lesbian students of color in a school for the Deaf on the East Coast.	3	18–20	Deaf	Lesbian	3/3 Proficient sign language users	Hearing family	Queer theory, disability theory, critical race theory, feminist theory, intersectionality theory, anti-oppressive education	Purposeful	Interviews	Action research
Eckhardt, 2006	United States	To gain information not currently available regarding the perceptions and experiences of individuals who are deaf related to HIV/AIDS and deaf culture.	2	25–40	Deaf	Gay Queer	Information not available	Information not available	Health belief model	Snowball	Survey, interviews	Constant comparative analysis
Gomez & Geneta, 2021	Philippines	Increase the awareness of the different sectors (i.e., education, health, and government) involved in ensuring the sexual health of young adults who are Deaf and LGBT+.	6	19–24	Deaf	2 Lesbian 2 Gay 2 Bi-sexual	6/6 Proficient sign language users	Information not available	Information not available	Purposeful	Interviews	Information not available
Grant, 2008	United States	To examine a small secretive group of women, deaf professional women, and their text as they told me their career life narratives in order to learn how they claimed citizenship in the workplace.	4	Information not available	Deaf	2 Lesbian 1 Bi-sexual 1 Transgender	4/4 Proficient sign language users	Information not available	Transculturation	Information not available	Interviews	Analytic induction
Kelly & Atcherson, 2011	United States	Assess perceptions of Quality of Life for older individuals with hearing impairment who have not consulted for services and their significant others who are in same-sex relationships versus those who are in different-sex relationships.	10	Mean age 71.56	Hard of Hearing	5 Lesbian 5 Gay	0/10 Proficient sign language users	Hearing family	Exploratory study	Bulletin boards, online, word of mouth	Survey	Information not available
Klein, 2021	United States	Explore Deaf lesbians in the United States from 1945 to 2020, how they reacted to and experienced their stigmatization and marginalization during that time period.	24	65–75	Deaf	Lesbian	24/24 Proficient sign language users	9 Deaf families 15 Hearing families	Crip theory, stigma theory, intersectionality theory	Snowball	Interviews	Grounded theory
Mallinson, 2004	United States	Describe the experiences of a subpopulation at a high risk for HIV/AIDS: deaf gay men.	5	24–49	Deaf	Gay	5/5 Proficient sign language users	Information not available	Information not available	Purposive	Interviews	Phenomenological analysis
Mauldin & Fannon, 2021	United States	Exploring the “Coming out” rhetoric in disability studies.	5	Information not available	Deaf	3 Lesbian 2 Gay	Information not available	5/5 Hearing families	Crip theory, Queer theory	Snowball	Interviews	Grounded theory
Mauldin, 2018	United States	Fill a theoretical and empirical gap in sociology by centering the voices of diverse Deaf individuals, specifically Deaf individuals of color who are also sexual minorities in the United States.	5	34–50	Deaf	3 Lesbian 2 Gay	5/5 Proficient sign language users	Hearing family	Emancipatory disability research	Snowball	Interviews	Line-by-line open coding
Michaels, 2015	United Kingdom	To pioneer and stimulate further research into the dynamics emerging in the relationship with those wider communities and organizations with which Deaf homosexuals engage.	15	Information not available	Deaf	Gay	15/15 Proficient sign language users	Information not available	Queer theory	Non-random	Interviews, questionnaire	Information not available
Miller, Biskupiak, & Kushalnagar, 2019	United States	To analyse the disclosure processes enacted by 31 LGBTQ students with disabilities at two universities in the southern United States.	2	Information not available	Hard of hearing	1 Bi-sexual 1 Transgender	1/2 Proficient sign language users	1 Hearing family 1 Deaf family	Intersectionality theory	Mailing lists	Interviews	Grounded theory
Pfau et al., 2021	Netherlands	To document Deaf LGBT history.	6	69–91	Deaf	1 Lesbian 3 Gay 1 Bi-sexual 1 Queer	6/6 Proficient sign language users	Information not available	Intersectionality theory	Snowball	Interviews, Online chat	Information not available
Phaneuf, 1987	Canada	To discuss some of the emotions, attitudes and general reactions toward homosexuality in deaf individuals.	4	20–23	Deaf	Gay	Information not available	Information not available	Information not available	Through Deaf associations	Interviews	Information not available
Schaad, 2015	United States	To explore the lived experiences and needs of LGBQ (Lesbian, Gay, Bisexual & Queer/Questioning) Deaf individuals within the Deaf community.	3	40+	Deaf	Lesbian	3/3 Proficient sign language users	Information not available	Participatory action research	Snowball	Questionnaire, focus group	Participatory action research
Schmitz, 2021	Germany	To describe this style, referred to here as “queer DGS,” from a Deaf-queer metalinguistic perspective.	8	20–50	Deaf, hard of hearing bilingual	Queer, lesbian, homosexual, gay, unlabeled, non-binary, genderfluid	8/8 Proficient sign language users	Information not available	Intersectionality theory, queer theory	Information not available	Focus group	Qualitative content analysis
Sinecka, 2008	Czech Republic	To examine the life experiences of Deaf gay people through the eyes of a young Deaf man growing up in the dominant hearing and heterosexual society in the Czech Republic.	1	24	Deaf	Gay	Proficient sign language user	Hearing family	Queer theory, theory of compulsory able-bodiedness	Information not available	Interviews	Life story
Ward, 2016	United States	To understand how Deaf students make sense of their college experiences in terms of their intersecting identities.	2	Information not available	Deaf	Lesbian	2/2 Proficient sign language user	Information not available	Tinto’s integration theory	Snowball	Interviews	Bottom-up analysis
Whalen, 2014	United States	To explore the transition experiences of Deaf Female-to-Male transgender individuals.	17	24–49 (mean age 36)	Deaf	Transgender	16/17 Proficient sign language users	Information not available	Feminist theory, grounded theory	Snowball	Interviews	Grounded theory
Willemse et al., 2009	South Africa	To give insight into the way his sexual identity is intersected with other identities, in particular with his Deaf identity, and how this “intersecting,” in the sense of how some identities are prioritized over others and how some are down played in the process, changes over time.	1	Information not available	Deaf	Gay	Proficient sign language user	Hearing family	Feminist theory, queer theory	Purposeful	Interviews	Biographic narrative
Zakarewsky, 1979	United States	To study the patterns of support among gay deaf men and lesbian deaf women.	2	25–26	Deaf	Gay	2/2 Proficient sign language users	Information not available	Information not available	Information not available	Interviews	Information not available

### Findings

Categories and themes are as follows: (1) institutional contributors to identity salience, with the themes: audism, religion, and ideas of success; (2) cultural moderators of institutional neglect, including the themes: language, lateral violence, and the hearing queer community; (3) resistance to double exclusion through community, with the themes: double exclusion, double acceptance, and romantic relationships; and lastly, (4) personal navigation of a contested body, including the themes: coping strategies, and confidence and acceptance. Significant quotes and theme descriptions are detailed in Table 3.

**Table 3:** Key quotes, descriptions, and sources to illustrate the synthesized themes under each category.

Category	Category description	Theme	Relevant studies	Quote
Category 1: institutional contributors to identity salience	This category details the structural influences enacted upon Deaf queer people, contributing to their connection, or lack thereof, to both Deaf and queer identities. Specifically, audism’s role, influencing parents to pursue oralist teaching pedagogy, religion’s homophobic dogma, and the concepts of “success” that these ideologies espouse, which are then imposed on Deaf queer people.	Audism	Celebrado, 2022; Cheslik & Wright, 2021; Conyers, 2008; Dunne, 2014; Grant, 2008; Klein, 2021; Mauldin, 2018; Whalen, 2014	“I didn’t call myself Deaf. I said I had a hearing problem. All of my life, I have been profoundly Deaf. My audiogram showed that I am way below the standard hearing level. The word ‘Deaf’ was banned. My parents told the school not to use the word ‘Deaf’.” (Klein, 2021)
		Religion	Cappotto & Rinaldi, 2016; Cherasaro, 2018; Conyers, 2008; Dunne, 2014; Eckhardt, 2006; Klein, 2021; Mauldin, 2018; Schaad, 2015; Whalen, 2014	“I was excommunicated from my Lutheran Deaf Church because I helped forming the GLBT Deaf club in the area. Even though my pastor knew I was gay 12 years ago.” (Conyers, 2008)
		Ideas of success	Beese & Tasker, 2022; Cappotto & Rinaldi, 2016; Conyers, 2008; Dunne, 2014; Grant, 2008; Klein, 2021; Mauldin, 2018; Michaels, 2015; Whalen, 2014	“I wouldn’t make myself marketable for a job. He said that if you can speak, you’re OK...They really drilled that into me. Therefore, if you can speak you will be successful in the world, and if you sign you are lesser. They really did tell me that over and over again.” (Mauldin, 2018)
Category 2: cultural moderators of institutional neglect	This category explains the ways in which Deaf queer people repel the audism and homophobia they face, primarily through connection with Deaf and queer cultures, respectively. The nuanced experiences within these cultures is discussed, where Deaf queer people face homophobia from the heterosexual Deaf community, and fetishization from the hearing queer community.	Language	Beese & Tasker, 2022; Cappotto & Rinaldi, 2016; Cherasaro, 2018; Dunne, 2014; Klein, 2021; Mallinson, 2004; Mauldin, 2018; Michaels, 2015; Sinecka, 2008; Whalen, 2014	“Jan is happy and proud now because, in his words, he found the ease and beauty of sign language communication and he identified himself with Deaf culture and the Deaf community. Jan said that he needed Deaf friends and sign language to be happy in his life. This is what keeps him alive.” (Sinecka, 2008)
		Lateral violence	Beese & Tasker, 2022; Cappotto & Rinaldi, 2016; Cherasaro, 2018; Conyers, 2008; De Abreu, 2015; Eckhardt, 2006; Grant, 2008; Klein, 2021; Mallinson, 2004; Pfau, 2021; Schaad, 2015; Schmitz, 2021; Ward, 2016; Whalen, 2014; Willemse et al., 2009; Zakarewsky, 1979	“Deaf people think it is important to be part of a group and have a group identity. When people find out, I am done having a group identity and I think that is worse than death. Do you know that?” (Klein, 2021) “[At Gallaudet College, a University for the Deaf] The people around me really didn’t treat me right, either. For example, I was walking past somebody and they sprayed me with pepper spray in the face. They knew. They also threw eggs and ketchup on my car. I never did anything to them. It was all because I’m a lesbian.” (Klein, 2021)
		Hearing queer community	Cappotto & Rinaldi, 2016; Cheslik & Wright, 2021; De Abreu, 2015; Eckhardt, 2006; Klein, 2021; Mallinson, 2004; Pfau et al., 2021; Phaneuf, 1987; Schaad, 2015; Schmitz, 2021; Willemse et al., 2009; Zakarewsky, 1979	“Louis, a young deaf man active in the gay deaf association in Quebec, says he and most homosexual deaf men he knows go to bars or clubs to meet other homosexuals despite the problems they encounter in the hearing gay world, such as difficulty of communication. If the deaf homosexual chooses not to ‘come out’ to other deaf homosexuals, the alternative choices are isolation or associations exclusively with hearing homosexuals.” (Phaneuf, 1987)
Category 3: resistance to double exclusion through community	This category recounts Deaf queer people’s experiences navigating dual identities, first negatively, where being Deaf and queer is a double stigma, and, then positively where a Deaf queer community is found and appreciated. Romantic relationships are also discussed, where the merits of hearing and Deaf partners are weighed.	Double exclusion	Beese & Tasker, 2022; Cappotto & Rinaldi, 2016; Celebrado, 2022; Cherasaro, 2018; Conyers, 2008; De et al., 2015; Dunne, 2014; Grant, 2008; Klein, 2021; Mallinson, 2004; Michaels, 2015; Schaad, 2015; Whalen, 2014	“There were times when people judged my ability because I am Deaf and gay. Some people ridiculed my signing and would say, ‘Parang malambot daw ako’ (I seem to be effeminate), and I would tell them that this is who I am.” (Celebrado, 2022)
		Double acceptance	Beese & Tasker, 2022; Cappotto & Rinaldi, 2016; Conyers, 2008; Dunne, 2014; Eckhardt, 2006; Klein, 2021; Mallinson, 2004; Michaels, 2015; Pfau et al., 2021; Schmitz, 2021; Willemse et al., 2009; Zakarewsky, 1979	“The deaf gay community is just so positive... it’s just been totally positive. I really enjoy it, it’s really a big thanks to them all as they’ve all helped me, with my confidence. I’m much more involved. I go out much more... it’s been amazing to have them as role models” (Beese & Tasker, 2022)
		Romantic relationships	Cappotto & Rinaldi, 2016; Cherasaro, 2018; Cheslik & Wright, 2021; Conyers, 2008; Dunne, 2014; Klein, 2021; Michaels, 2015; Pfau et al., 2021; Phaneuf, 1987; Sinecka, 2008	“‘Why not just date a Deaf gay man?’ At face value, this seems to be the easiest solution, but participants point to the myriad reasons why this is not always feasible. For one, geography plays an important role—Deaf gay men are scattered across the nation, preferring metropolitan areas that have accepting attitudes towards the Deaf and gay community. However, those who live in such areas are a small percentage of the overall Deaf gay population. Responsibilities such as family, work, friends, and ‘home’ were among the reasons why participants reported living where they do. Another reason is simply a matter of preference. Some participants felt that dating or finding sexual encounters within such a small pool poses a limited number of choices, while the hearing gay community is vast in comparison. As such, some participants felt that the options for potential suitors were more varied despite the risk of continual rejection. A majority of participants iterated that ‘playing’ instead of dating within a small Deaf community runs inherent risks among them, possibly being easily ‘outed’ or a partner would easily find out if one was partaking in activities outside the realm of agreements made as a couple.” (Cheslik & Wright, 2021)
Category 4: personal navigation of a contested *body*	The final category takes a personal approach to dual identities, and how institutions, cultures, and communities impact the individual navigation of identity. Coping strategies that Deaf queer people enact are described. Lastly, the resilience of this population is shown in their acceptance of both identities.	Coping strategies	Beese & Tasker, 2022; Cherasaro, 2018; Cheslik & Wright, 2021; DeAbreau, 2015; Grant, 2008; Klein, 2021; Mauldin, 2018; Michaels, 2015; Miller, Biskupiak, & Kushalnagar, 2019; Pfau et al., 2021; Schaad, 2015; Schmitz, 2021; Sinecka, 2008; Zakarewsky, 1979	“Sometimes I feel that I was forced to speak, that I was forced to be a hearing person. I had to be involved in doing things that way, in that [hearing] culture.” She goes on to talk about obtaining a cochlear implant at the age of eighteen in an effort to speak with her family. “[The implant] had a lot to do with the conflict that was going on inside of me, all the conflicting desires. A priority for me was to try and communicate better with my family.” (Mauldin, 2018) “I would never discuss about gay/lesbian experiences right here [at Gallaudet College]. Keep quiet, keep going on with my life. At night, we would get together and go to bars or house parties. But during the daytime, I would be a ‘good girl’. I didn’t want to get in trouble, didn’t want to get fired. I liked my job, I liked working with Deaf people, and I had a nice social life. Unlike other places, with hearing coworkers. I’d rather be working at Gallaudet. That’s why I had to be careful.” (Klein, 2021)
		Confidence and acceptance	Beese & Tasker, 2022; Cherasaro, 2018; Conyers, 2008; Dunne, 2014; Grant, 2008; Mauldin, 2018; Pfau et al., 2021; Sinecka, 2008; Whalen, 2014; Willemse et al., 2009	“I had two identities that I came to accept. For a long time, I repressed both of these. But I became a stronger person and I stood up for what I believed in. I came out to my parents around the same time I told them I identified as Deaf. Around nineteen or twenty years old. I told my dad I was gay around twenty or twenty-one. That’s such an age of not knowing. Then you know, you just know that this is what I am, this is my perspective, this is where I stand. So when my mom says things to me, I cannot cower and just accept it, I can challenge it. Before when that would happen, I kept quiet. I was silent. It was hard for me to get the words out. The older and stronger I became as a person, the more I stood up for myself... I wasn’t weak and just a follower of whatever people said. Just because my mom and dad say it is so doesn’t mean I’m just going to follow it, that they know best. I wasn’t just going to just do what they say [...] I felt happy afterwards. My best year was when I was twenty-two. That was the year after I came out, I felt comfortable, I was thrilled. [...] I went through a lot of introspection, I analysed myself, figured out who I was, what I wanted to do, I figured out myself and I told people about it. I said this is who I am. If you can’t accept it, then it’s your problem” (Mauldin, 2018)

### Category 1—“Well, number one, there’s no support” institutional contributors to identity salience

#### Audism

Audism was described in eight studies, with the participants’ life experiences irrevocably influenced by the dominant system of the hearing world. Parental audism was common, either passively or actively upholding deafness as subordinate, and supporting medical “fixes” (Mauldin, 2018). Participants often felt lonely, abandoned, and disconnected from their families, where “conversations were surface-level” (Cherasaro, 2018). The most salient product of audism was an oralist pedagogy, where “signing was forbidden” (Klein, 2021). This excluded participants from social interactions and an adequate education, participants reporting: “when I asked, ‘what does that mean?’ they would just say ‘never mind’” (Cappotto & Rinaldi, 2016). These types of discussions situated deafness as a deficit, with some participants aligning themselves with the oppressive system of audism leading to internalized beliefs about the signing Deaf community. These beliefs included “hearing people are more.... er intelligent” (Beese & Tasker, 2022). Some participants did not socialize within the Deaf community because “[they] thought [they were] better than them” (Klein, 2021). Participants reported that this audist pedagogy was enforced by audiologists, speech pathologists, and general practitioners (Beese & Tasker, 2022; Cappotto & Rinaldi, 2016; Dunne, 2014; Klein, 2021; Mauldin, 2018).

A salient sub-theme was a lack of medical and identity-related information in adulthood; audism limiting d/Deaf people’s access to, and comfort within, medical institutions (Cappotto & Rinaldi, 2016; Cheslik & Wright, 2021; Dunne, 2014; Klein, 2021; Mallinson, 2004; Michaels, 2015; Pfau et al., 2021; Phaneuf, 1987; Schaad, 2015; Willemse et al., 2009; De et al., 2015). One participant did not learn the term lesbian until adulthood, replying “le... what? Would you write it down?” (Pfau et al., 2021). This was often perpetuated by schools, limited discussion about sexual identities leading to participants gleaning information from gay social networking sites (Cheslik & Wright, 2021; Klein, 2021). Participants also espoused uninformed and risky sexual behaviors, some having unprotected anal sex and others believing that “taking a bath is enough for safe sex”, increasing the risk of contracting HIV/AIDS (Gomez & Genata, 2021; Mallinson, 2004). As a response to this audist neglect, the d/Deaf queer community united and participants shared “information with each other” to nullify the medical English that “many Deaf don’t understand” (Mallinson, 2004; Michaels, 2015).

### Religion

Religion was the vehicle for prejudice in nine studies, where participants described their queer and d/Deaf identity as a “sin”, and detailed experiences of exclusion and violence by religious institutions (Conyers, 2008; Dunne, 2014). Like audism, religious oppression was often perpetuated by family. A father said, “all deaf people have the devil inside of them”, a preacher declared that “that god would make [them] hearing”, and a brother attended a “religious revival” to cure deafness (Mauldin, 2018; Schaad, 2015). Many participants felt strong internalized homophobia, aligning themselves with discriminatory institutions, that directed hate inwards or at other members of the Deaf queer community, taking pride in being “straight acting”, not signing in “a gay way”, and having disdain for those who proclaim “I’m gay, I’m gay, I’m gay” (Beese & Tasker, 2022; De et al., 2015).

### Ideas of Success

The concept of success was a primary motivator for discriminatory behaviors in nine studies. Parents often prioritized normative expectations, such as “independence”, “marketability for jobs”, and “[straight] marriage” (Dunne, 2014; Mauldin, 2018; Whalen, 2014). One parent was painfully clear in their expectation of institutional normativity, saying “if you can speak you will be successful in the world, and if you sign you are lesser” (Mauldin, 2018). In contrast, some participants found success in their own ways: “I’m deaf, teaching mathematics and ASL (American Sign Language) [and] have a great smile. I am successful and proud of myself”, and that “I founded many things, I established events that still run [...] students respected me. [....] I was a good leader [... and] I made breakthroughs for the gay and lesbian community” (Conyers, 2008; Klein, 2021). These participants shed normative expectations and found pride and “success” within their communities, despite discrimination and institutionalized neglect.

### Category 2—“Deaf is Deaf and that’s all that matters” cultural moderators of institutional neglect

#### Language

Connection through sign language united the participants in ten studies, as some participants gained access into a vibrant Deaf community and gained confidence in their identity as Deaf: “I became so proud after I learned what my name was, and I learned sign, and then later I learned [...] who I was” (Mauldin, 2018). Sign language was integral to their identity as a Deaf individual, where culture “really is about sign language” and sign language represents an important separation from the hearing world, and language deprivation: “it shows strength and acceptance of who you are [...] when Deaf people are together they don’t have to struggle with understanding language” (Michaels, 2015). Adoption into the Deaf community often accompanied a Deaf role model: “if she had not taught me how to sign [...] I would not [...] have realised how cool it is to be Deaf. She gave me the birth and now, I am a happy man” (Sinecka, 2008). This rebirth as a Deaf person, fully embodying language as an identity, signifies a rejection of oralism and embrace of Deaf culture.

### Lateral violence

Violence was a common experience within and outside the Deaf community, including assault, rape, molestation, and incest (Beese & Tasker, 2022; Celebrado, 2022; Conyers, 2008; Dunne, 2014; Klein, 2021; Pfau et al., 2021; Whalen, 2014; Willemse et al., 2009; De et al., 2015). Participants felt more susceptible to hate crimes in the hearing world due to their deafness (Beese & Tasker, 2022); however, others felt their deafness was an asset in this context, finding it easier to ignore mocking or rejection in public (Cherasaro, 2018). In comparison, while the Deaf community could be an oasis for participants, its insular nature was also a source of fear for participants in 16 studies, many participants reporting experiencing lateral violence because of their queer identity. The value of information-sharing made it “harder [...] to come out in the deaf community. Because once you come out, everyone knows” (see Table 3). Participants refused interpreters in medical appointments “because they are so dreadfully frightened that their secret will get out to the deaf community” (Beese & Tasker, 2022; Eckhardt, 2006). At times, this gossip was participants’ first exposure to queerness, framing the subject in a negative light from conception: “I remember my mom signed lesbian [...] my mom showed a negative reaction. Her facial expression showed it all” (Klein, 2021). This heterosexual standard was magnified by social class, where Deaf queer people who had Deaf family felt a pressure to uphold the prestigious “Deaf-of-Deaf” image, where to be queer was to “be embarrassed [...] I didn’t want to embarrass my family [...] I had to be careful [...] because people knew me” (Klein, 2021).

Social class was a significant sub-theme, where some participants felt “that [they had] been treated differently because [they] didn’t grow up using sign language” (Beese & Tasker, 2022; Cappotto & Rinaldi, 2016; Cherasaro, 2018; Cheslik & Wright, 2021; Dunne, 2014; Grant, 2008; Klein, 2021; Michaels, 2015; Ward, 2016; Whalen, 2014). Language accounted for a large portion of this separation, where some participants struggled to “slow down [their] signing” (Ward, 2016). The result of this was feeling “a little bit like a second-class citizen”, and feeling that “if [they] had grown up deaf and signing, [they would] be more confident [and] comfortable in big groups” (Beese & Tasker, 2022; Michaels, 2015).

### Hearing queer community

Due to lateral violence from the Deaf community, participants in 12 studies “reported a preference for hanging out with hearing lesbians [and queer people]” (see Table 3). This was somewhat of a “stop-gap”, before beginning to “miss the Deaf world” (Pfau et al., 2021). Experiences within the hearing queer community were mixed, some reporting that it “is not yet sufficiently sensitised to the problems of deafness”, whereas others “feel a strong sense of belonging” (Schmitz, 2021; Zakarewsky, 1979). Experiences within the hearing queer community were moderated by environment, where participants would “communicate with paper and pen” in loud club environments, or hide their deafness on dating apps (Pfau et al., 2021). Some faced rejection when transparent about their Deafness, potential suitors telling them “Never mind, I don’t want to waste my time on limited conversations” (Cheslik & Wright, 2021). Contrarily, participants also reported fetishization by hearing queer people, some hearing people “loved sleeping with Deaf guys and felt that they were better in bed,” participants stating that “I guess with my voice, I was not ‘disabled’ enough, not exotic enough, for [them] [...] he wanted the novelty of having sex with Deaf person”(Cheslik & Wright, 2021; Mallinson, 2004).

### Category 3—“I think the deaf gay community feels more safe when we’re all together” resistance to double exclusion through community

#### Double exclusion

While some participants reported no discrimination based on either identity (Conyers, 2008), 13 studies reported homophobic or audist targeting within each community. Ward’s (2016) study mentioned a “Deaf plus” label that forced subgroups within the broader Deaf community, and Schaad (2015) framed these dual statuses as “double stigma[s]”, increasing feelings of isolation (Michaels, 2015). Deaf lesbian elders felt conflict over where to live; “Delaware may be a better place for me. They have a strong hearing lesbian community there. [...] there’s a lesbian retirement community [...]. The problem is that there’s not many Deaf people. I’m tired of moving so much” (Klein, 2021). Deaf queer people were also discriminated against in the workplace, with one participant experiencing a dual audist and homophobic incident at work: “I can tell that they would call me bakla (gay). I can read it through their lips that they say, ‘bakla, bakla, bakla’” (Celebrado, 2022). Exclusion came from the hearing heterosexual society, but also within the Deaf community. For example, students at Gallaudet College were “sprayed [...] with pepper spray in the face. [...] They also threw eggs and ketchup on my car. [...] It was all because I’m a lesbian” (Klein, 2021). These incidents of exclusion foster the desire for a Deaf queer community, where both identities are understood and accepted.

### Double acceptance

Participants in 12 studies reported favorable experiences within the Deaf queer community, stating that these communities “opened up new possibilities of open communication and authenticity”. Older Deaf queer participants took pride in establishing these conventions, clubs, and conferences (Pfau et al., 2021; Zakarewsky, 1979). Characteristics of these communities included drag and language adaptations. For example, “in the Deaf community my name is John. [...] In the gay and lesbian community I am called Queen.” (Willemse et al., 2009). Gay Sign Variation was important, including code language such as “‘are you one?’” [...] to identify others and prevent rumours” (Klein, 2021; Michaels, 2015). Information sharing was integral: “there’s more knowledge about safe sex because of sharing information”. However, this was a double-edged sword, some participants noted the community was “always in groups, backstabbing each other. Bitching about each other” (Beese & Tasker, 2022). Overall, participants felt that they “would have never been the person [they are] today without [...] interaction with the deaf and gay communities” (Beese & Tasker, 2022).

### Romantic relationships

Romantic relationships were varied, with some studies suggesting that Deaf queer people preferred hearing partners (Cherasaro, 2018; Cheslik & Wright, 2021; Dunne, 2014; Klein, 2021; Michaels, 2015; Pfau et al., 2021; Phaneuf, 1987), and some asserting the opposite (Cappotto & Rinaldi, 2016; Conyers, 2008; Sinecka, 2008). Despite relatively easier accessibility, the main contention surrounding relationships with hearing people was language: “unless the woman knew some ASL, she would not have been able to communicate” (Cherasaro, 2018; Cheslik & Wright, 2021). The use of technology alleviates this issue somewhat, “apps provid[ing] a way to start a conversation” (Cheslik & Wright, 2021; Dunne, 2014). However, in person, participants felt “behind in the conversation” where their partners “try and help but [they] never feel it’s equal” (Michaels, 2015). Dating interpreters seemed a good choice due to their fluency in sign language and awareness of Deaf culture, however, their professional boundaries with the Deaf community limited “socialisation and fraternisation” with their partner’s friends (Cherasaro, 2018). Dating a Deaf queer individual was also reported to be a cathartic experience; “I know what it feels to [use] hands to speak and love. [...] I am with her forever”; however, there were risks dating within such small communities (Cheslik & Wright, 2021; Conyers, 2008).

### Category 4—“I am deaf and gay. Proud of who I am. Fuckers can fuck off”: personal navigation of a contested body

#### Coping strategies

The most salient coping strategy, reported by participants in 14 studies, was masking. One participant aptly stated, “if you want to be loved, you have to adapt”, meaning that to be Deaf and queer necessitated hiding one or both identities throughout life (Schmitz, 2021). Participants varied in their levels of awareness of this being maladaptive (see Table 3; Beese & Tasker, 2022; Schmitz, 2021). Participants who internalized their homophobia often projected their insecurity onto prideful queer people: “I’m not completely proud and waving my hands about it—no. I try to be straight” whereas others were painfully aware of the double life they led, stating “at night, we would get together and go to bars or house parties. But during the daytime, I would be a ‘good girl’” (Beese & Tasker, 2022; Klein, 2021). Some participants described oralism as a form of masking, modifying their true Deaf self to appease a hearing world; “I really wanted to sign but would just sigh and go ahead and use my voice” (Mauldin, 2018). This masking prevented participants from experiencing life, one participant saying they: “do not enjoy going to—live concerts and things like that [...] I cannot hear things properly and then I feel excluded” (Miller et al., 2019). A large motivation for sexuality masking was privileges in the Deaf community, such as working in a Deaf space. One participant hid her sexuality for “six to seven years”, placing her Deaf identity above her queer one (Grant, 2008; Klein, 2021; Schaad, 2015). Other coping strategies included being silent about access needs and negative experiences (Cappotto & Rinaldi, 2016; Dunne, 2014; Grant, 2008; Klein, 2021), educating hearing and Deaf communities about accepting dual identities (Celebrado, 2022; Conyers, 2008), staging protests and conducting advocacy work (Klein, 2021; Pfau et al., 2021), and believing when young that one would become hearing, straight, or cisgender with time (Whalen, 2014).

### Confidence and acceptance

Participants in 10 studies expressed a deep satisfaction with, and embraced, their dual identities, becoming “a stronger person [...when they] stood up for what [they] believed in”, telling people “this is who I am. If you can’t accept it, then it’s your problem”. The following statements are indicative of the peace that comes with this acceptance: “I am a proud trans-person” (Grant, 2008) and “Ok, so I was lesbian. I was also deaf. I was a deaf lesbian. I was a deaf lesbian in love, in love with a deaf woman. That was clear. That was nice” (Pfau et al., 2021). Participants credited this acceptance either to time, language, or a “guide,” who increased affiliation and exposed them to each culture (Mauldin, 2018).

## Discussion

The current study has synthesized qualitative literature about experiences of d/Deaf queer people, providing previously under-researched insight into the identities of a doubly marginalized population. The MMDI framed the findings, with the model expressing how identities change throughout time and environment (Jones & McEwen, 2000). Participants’ identity salience was influenced by institutional neglect, cultural moderators, community access, and personal factors. The categories and themes give a broad insight into the ways dual oppression is perpetuated by systems, and infiltrate communities in ways that at times diminish connection, leading to a fracture where both identities must be present to be valued. Further, the findings highlight the importance of shared language and adaptive coping strategies within institutionalized subjugation.

### Deaf identity salience: categories one and two

Deaf identity was held closely to one’s core, oftentimes acting as the most salient component of participants identities. This was in large part due to category one, institutional influences, and category two, cultural influences. Identity salience within the MMDI is affected by contextual influences, depicted in the model as an all-encompassing circle surrounding the atom shape of each identity (Jones & McEwen, 2000). These contextual influences include “family background, sociocultural conditions, current life experiences, and career decisions and life planning” (Jones & McEwen, 2000, pg. 410). Participants’ Deaf identities were irrevocably influenced by their sociocultural conditions: namely, the audism they experienced as described in category one. The discrimination participants faced resulted in a greater appreciation of this identity category. Participants stated that their Deaf identity was more salient due to their “access to communication”, and that they were Deaf “100% of the time”. In contrast, participants reported their queer identity as being expressed less frequently, happening “at weekends with friends” or solely between “two men [...] or two women together” (Beese & Tasker, 2022; Michaels, 2015). Past researchers have reported similar outcomes investigating (e.g., (James & Woll, 2004), as Deaf and Black participants have also reported their Deaf identity to be more salient due to the hearing world’s influence upon their existence. However, these correlations must be considered with caution. Researchers should ensure they do not to use racial boundaries as opportunities for analogous comparisons in marginalized groups and avoiding the assumption that to be Deaf queer is inherently positioned as separate from being a person of color, given that many participants in this study were non-white themselves (Frederick & Shifrer, 2019). Broadly, identity literature supports the proposition of oppression fostering a stronger connection to a singular identity, and the relegation of others (Gibson, 2018).

Participants displayed transitional meaning-making capacity according to the MMDI’s 2007 revision, where audism was the catalyst for their core Deaf identity (Abes et al., 2007). This transitional meaning-making capacity highlights the importance of social identities, where participants identify the limits of stereotypes upon their identities and become frustrated by the audism they experience while still possessing an inability to combine and integrate multiple identities (Abes et al., 2007). Unlike other identity categories, being Deaf is not internally defined from birth; it is dependent upon schooling, language, family environment, and exposure to other Deaf people (Ahmad et al., 2002). Deaf identities formed and articulated in the sphere of external influence made the cultural impact described in category two especially relevant for Deaf identity salience. Similarly to participants in Ahmad et al. (2002), Chapple et al. (2021), Skelton and Valentine (2003), and Smiler and McKee (2007), the participants in this study articulated the importance of language for their Deaf identities to flourish, and therefore take precedence: “I use sign language every day [...] so therefore my Deaf identity is more important to me” (Michaels, 2015). Language was conceptualized as an antidote to discrimination and trauma: with access to a signed language, Deaf identity salience was allowed to flourish and was consistently recounted as integral to one’s Deaf identity. Researchers have previously indicated the importance of sign language acquisition to decrease the precedence of “cognitive delays, mental health difficulties, lower quality of life, higher trauma, and limited health literacy” (Clark et al., 2016; Hall, 2017, pg. 961; Humphries et al., 2014; Meier, 1991; Senghas & Monaghan, 2002). This study furthers the call for sign language, identifying it as not only important for educational, health, and cognitive outcomes, but also for positive and robust identity development^2^.

### Queer identity salience: categories three and four

Some participants described their queer identities as being closer to their core, but predominantly Deaf identities took precedence. Participants’ queer identities were overshadowed by their double exclusion described in category three; hence, they employed masking techniques (category four) to survive within the Deaf community. In effect, participants’ queer identities were consciously relegated to the farthest orbit from their core, prohibited from being internalized as important and as salient as their Deafness. For participants, the discrimination and daily marginalization that accompanies Deafness made the pride and enactment of Deaf values more important to withstand audism. This can be likened to Dharani’s (2024, pg. 3) research on intersectionality, where the “dance between oppressors and the marginalised” necessitates the creation and value of marginalized identity categories. Participants described their queer identity as “just a part of [them]” whereas they “are” a Deaf person (Michaels, 2015). The relegation of queerness to behavior is remarkably like that described by Foucault before the categorization of homosexuality in the 19th century, and solidifies the participants wish to view their queerness as contextual, situational, and inconsistent in comparison with their Deaf identities (Foucault, 1978).

This simplification and relegation of queerness to the external edges of identity reflects the double exclusion described in category three. The importance of community to Deaf identity, the homophobia within this community, and the relative importance of Deafness serve as additive influences on participants’ queer identities. The combination of these factors forces the participants to reconsider their queer identity’s importance, whether conscious or unconscious, and enact coping strategies such as masking, as described in category four, to hide or downplay their queerness in service of their Deaf identity. It has been suggested by Skelton and Valentine (2003), Wardle (2017), and Zangas (2005) that the Deaf community’s isolation from normative information streams excludes them from the continual changing tide of public opinion on political issues, providing them with little engagement with movements for queer acceptance and civil rights. However, Wardle (2017) also notes that improvements to accessibility for the Deaf community have led to greater engagement with and awareness of political movements. This is reflected in Zangas’ (2005) conclusions that overall, the Deaf community has moderately positive attitudes toward queer people, but also potentially reflects that acceptance is subject to d/Deaf queer participants’ minimization of their queer identities.

### Dual identity salience

Both d/Deaf and queer identities are markedly different to those usually analysed through the MMDI; they are both largely invisible—making them uniquely susceptible to influence from their respective communities and internal values (Jones & McEwen, 2000). Neither identity is immediately noticeable to the general public; unlike race, gender, or in some cases, religion. Within this context, the revised MMDI helps to frame and analyse these dual identities by categorizing the level of filtering through which participants understand their invisible identities and thus, how much contribution external influence has on their overall conceptualizations of identity (Abes et al., 2007). The participants in this study showed mostly formulaic meaning-making, with brief forays into transitional understandings of identity (Abes et al., 2007). Participants were often unable or unwilling to draw connections between their dual identities and operated largely with both identities held separately within their conceptions of self. This inability to consolidate identities was specifically noted by Schaad (2015) and Dunne (2014), who stated that there was a “lack of awareness about the complexity of multiple identities”, and participants lacked the “vocabulary [...] to discuss the negotiation of identities”.

Research by Patton and Simmons (2008) highlights this formulaic conception of identity, where their Black and lesbian participants expressed their identities separately due to the difficulty of finding a community where both identities could be accepted together. Further, Miller’s (2018) work with queer and disabled college students suggested that the most common conceptualization for dual identities was oppositional, participants negotiating their identities as separate threads. Miller (2018) posited that this was a self-protective strategy, “to escape identifying with the weight of multiple forms of oppression, at least temporarily” (pg. 340). Here, the importance of category three—double acceptance—is evident. The ability for d/Deaf queer people to form communities is integral to foster a more intersectional and nuanced conceptualization of dual identities (LeBlanc & Tully, 2001).

### Strengths, limitations, and implications

The d/Deaf queer population is thoroughly under researched. This study’s ability to synthesize previous literature on the topic is considered a strength, and can provide a comprehensive view of what is known, ensuring that future research has a solid foundation to build on. Further, this study exemplifies the positive influence of language, community, and peer role models on positive identity development. With the rise of technology and the significant decrease in community events and spaces for the Deaf community, there is a fracturing of these integral spaces for connection (Pfau et al., 2021). Further, the push for integrated schooling reduces opportunities for d/Deaf peer integration and connection (Bat-Chava, 2000). This study reinforces the importance of these connections and urges community leaders and policy-makers to ratify appropriate and therapeutic spaces for Deaf connection and identity development.

This is the first systematic review about Deaf queer people’s identity experiences thus, the findings uniquely synthesize these minority experiences. Further, this study answers the call for Deaf Studies to become more inclusive, investigating the heterogenous d/Deaf community with care to establish unique experiences and variances within this group (Smiler & McKee, 2007). The studies included were of broad geographic origin, and ensured that a range of experiences were captured and synthesized in the results. While most included studies were of American origin (the United States, Brazil, Canada), there was significant input from Europe (Italy, the Netherlands, Germany, Czech Republic, the United Kingdom). However, there were no studies from Australia, only one from Africa (South Africa), and two from Asia (the Philippines). The results showed broadly comparable experiences of audism and homophobia. It would be beneficial for further studies to explore further the possible nuances of Asian, Australian, and African d/Deaf queer communities. The age range represented in studies was also broad, spanning from 18 to 91. This ensured that possible history and age-graded events were captured, especially where both identities were subject to vast changes in treatment and public perception in the 20th and 21st centuries; specifically during the HIV/AIDS crisis of the 1980s and 1990s, and the educational pedagogy shifts of the 1950s to 2000s (Phaneuf, 1987; Zakarewsky, 1979; Kennedy & Buchholz, 1995).

Despite the strengths of this study, there were notable limitations. This study aimed to provide as broad a review as possible, to capture existing knowledge. For this reason, we chose to include all studies falling under a queer umbrella term (i.e., all sexual orientation and gender labels). Future studies and reviews may benefit from focusing on individual sub-groups to learn further specific and nuanced information about different experiences for each group. Particularly, the current research only included two studies with transgender or non-binary participants, whose experiences may diverge significantly from other queer experiences (Whalen, 2014; Ware & Marshall, 2014). The scope of this study did not extend to the experiences of multicultural and multilingual Deaf people. However, some participants certainly fell outside the dominate cultural-linguistic paradigms (e.g., Klein, 2021 in particular). As such, we acknowledge the unavoidable exclusion of multicultural and multilingual Deaf people in the study as a limitation. The current study’s sample mostly consisted of participants with hearing parents. While this is proportional to the population, further studies may seek to investigate positive experiences of language acquisition for d/Deaf people, including those with Deaf families. Last, this study was limited by the studies available—many of which were dissertations and theses. In an already under researched topic, it is integral for future peer-reviewed studies to be published, to ensure a robust information base.

## Conclusions

The current study investigated the experiences of d/Deaf queer people, and how these dual identities shift. The findings elucidated four categorical influences on identity salience: institutional, cultural, community, and personal. The institutional structures of audism and homophobia, enacted upon d/Deaf queer people, limit their opportunities to embrace their dual identities and find intersectionality. The antidotes to these harmful structures range from personal (masking one or both identities) to community based (by finding other Deaf queer people to engage and share experiences with). The findings advance current literature about the heterogenous d/Deaf community and highlight the importance of uplifting this doubly marginalized group.

## Footnotes

1
*Lesbian Gay Bisexual Transgender Queer (or Questioning) Intersex Asexual (or Ally), Men Who Have Sex with Men, Gender and Sexually Diverse (*  Glazier, 2014*).*

2 The debate surrounding signed languages has only become more complex with the increased prolificity of Cochlear Implants (CIs). Some Deaf community members liken the devices to tools of cultural genocide, while others recognize their benefits when used in conjunction with a bilingual education approach. One participant in Whalen (2014) weighed in on the issue, stating that CIs reduce the space between hearing and Deaf worlds, effectively traversing the boundaries of both. For Deaf separatists, they argue, the defence against an audist world is to create robust and distinct Deaf spaces, for which an implanted Deaf person may be conceived as “belittling the power that they have managed to take back” (Whalen, 2014). Discourse surrounding CIs is complex, and exemplifies the rejection of audism and dealignment with medical technologies that characterizes a Deaf identity.

## Preregistration

This study was not preregistered.
